# Supplementary figures and images for: Dark Web Marketplaces and COVID-19: before the vaccine
Source: EPJ Data Sci. 2021 Jan 21;10(1):6. doi: 10.1140/epjds/s13688-021-00259-w (PMC7819623; doi:10.1140/epjds/s13688-021-00259-w)

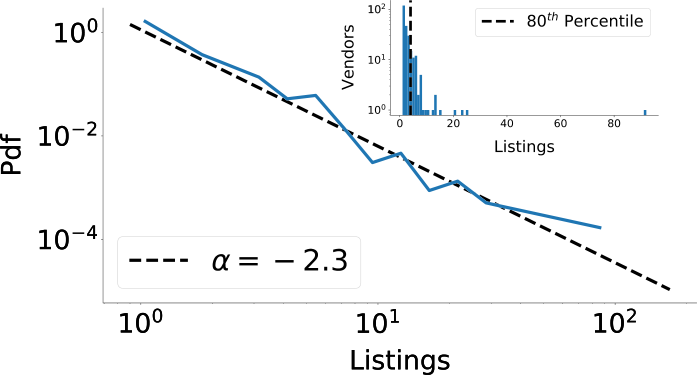

Supplement: Supplementary file 1 — Probability distribution function (Pdf) for the number of listings per vendor. The power law fit results in an exponent of −2.3. In inset, the histogram of the number of listings per vendor, with a vertical line showing the 80th percentile [file 13688_2021_259_Fig7_HTML.png]

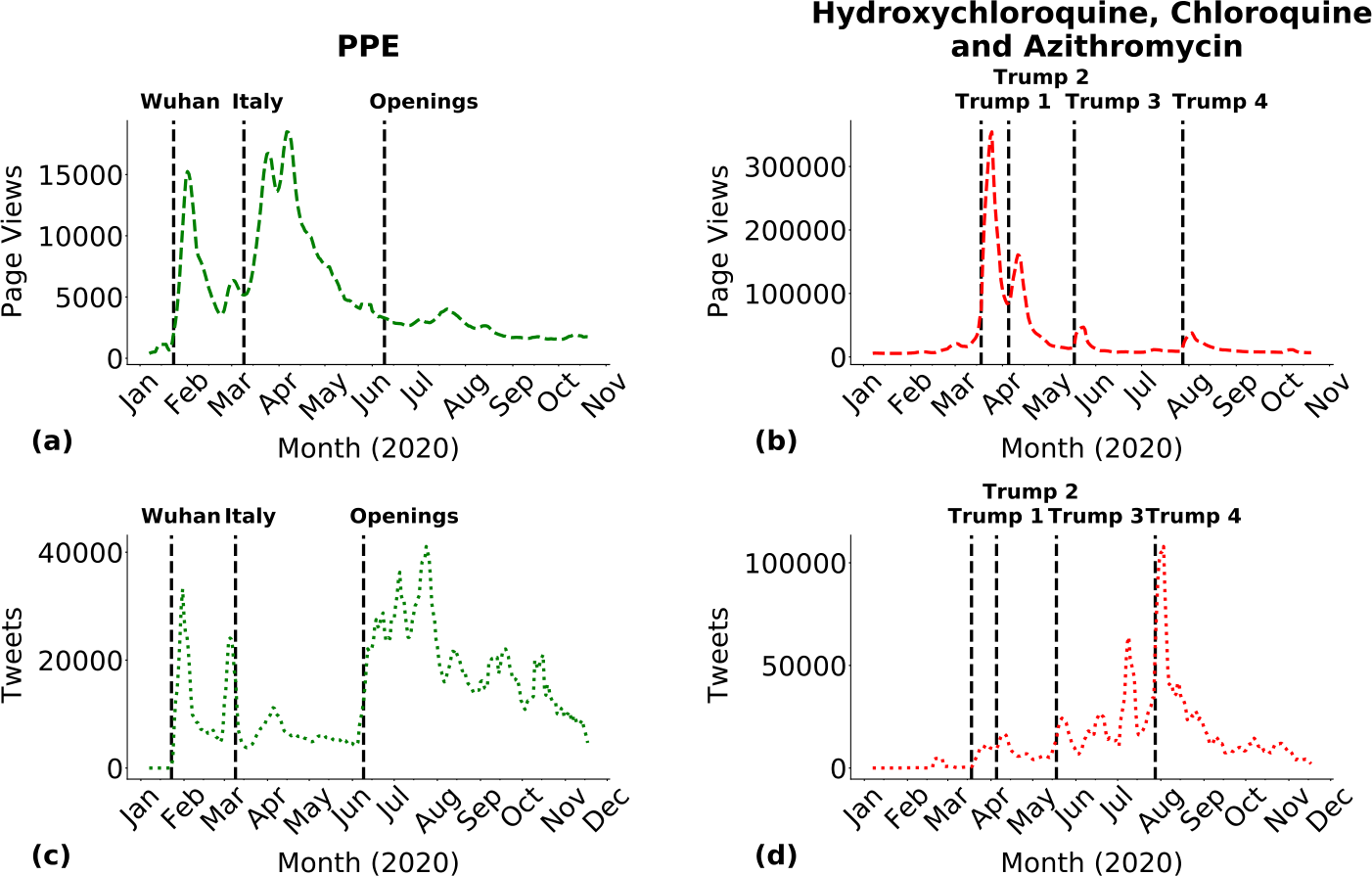

Supplement: Supplementary file 2 — Time evolution of the active COVID-19 specific listings in the guides on scamming category. (a) Seven-days rolling average of these observed listings at a given time. Black dashed vertical lines corresponded to significant COVID-19 world events, see Appendix C. (b) Seven-days median price with 95% confidence interval for these observed listings [file 13688_2021_259_Fig8_HTML.png]

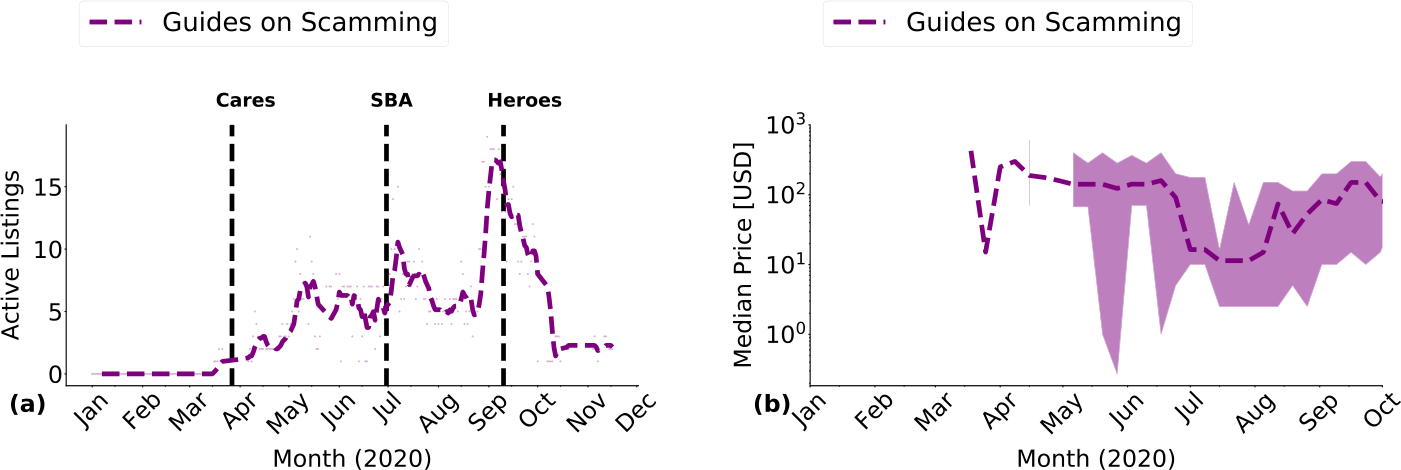

Supplement: Supplementary file 3 — Wikipedia page visits for pages relative to (a) PPE, (b) hydroxychloroquine, chloroquine and azitrhomycin. Number of tweets mentioning (c) PPE, (d) hydroxychloroquine, chloroquine and azitrhomycin. Panels (a) and (b) corresponds to Fig. 5(a) in the main text, while panels (c) and (d) to Fig. 5(b). The main difference between these panels and Fig. 5(a) and (b) is the linear scale on y axis [file 13688_2021_259_Fig9_HTML.png]
